# Prevalence of Stroke in Spain and Its Impact on Quality of Life: Socioeconomic Inequalities and Access to Rehabilitation

**DOI:** 10.3390/healthcare13091075

**Published:** 2025-05-06

**Authors:** Ismael García-Campanario, Maria Jesus Vinolo-Gil

**Affiliations:** 1 Department of Medicine and Surgery, Faculty of Medicine, University of Cádiz, 11003 Cádiz, Spain; 2 Research Group PAIDI UCA CTS391, University of Cádiz, 11003 Cádiz, Spain; 3 Department of Nursing and Physiotherapy, University of Cádiz, 11003 Cádiz, Spain; mariajesus.vinolo@uca.es; 4 Institute for Biomedical Research and Innovation of Cádiz (INIBICA), 11009 Cádiz, Spain; 5 Rehabilitation Clinical Management Unit, Interlevels-Intercenters Hospital Puerta del Mar, Hospital Puerto Real, Cádiz Bay-La Janda Health District, 11006 Cádiz, Spain

**Keywords:** stroke, prevalence, rehabilitation, quality of life, physical activity, socioeconomic factors

## Abstract

Stroke is a cerebrovascular syndrome due to a sudden interruption of blood flow to the brain that causes transient or permanent damage. Despite advances in the field of medical science, stroke is still common and continues to have a significant effect on quality of life. Objective: The objective of the study was to analyze the prevalence of strokes in Spain, considering differences in sociodemographic factors, functional limitations, and access to rehabilitation, with special attention to sex-related disparities. Methodology: A cross-sectional study was conducted using data from the latest European Health Survey in Spain carried out between 2019 and 2020 on a total of 22,072 people. Individuals over 16 years of age with a medical diagnosis of stroke were selected for this study. Sociodemographic variables, self-perception of health, limitations in daily activities, level of physical activity, and access to rehabilitation treatment were analyzed. Descriptive measures and inferential tests were applied for statistical analysis. Results: The prevalence of strokes occurring in Spain was estimated at 2.02%, which is lower than European figures. Men tend to have strokes at younger ages (50% between 36 and 73 years), while women report a poorer quality of life after the incident. Most patients had not received rehabilitation services in the last year. Conclusions: Given the aging population, it is essential to reinforce prevention, early detection and rehabilitation therapies to improve quality of life and reduce the burden of care.

## 1. Introduction

A stroke (cerebrovascular accident) is defined as a clinical syndrome caused by a sudden disturbance in cerebral blood flow that leads to focal or global neurological impairment. According to the International Classification of Diseases (ICD-10), strokes are classified primarily as ischemic stroke (ICD-10 code I63), accounting for approximately 80% of cases, and hemorrhagic stroke (ICD-10 code I61), representing around 20% [1]. Ischemic stroke results from an obstruction of blood flow, typically due to thrombosis or embolism, while hemorrhagic stroke is caused by the rupture of a blood vessel, leading to intracerebral bleeding [2,3]. In Spain, approximately 120,000 people suffer a stroke and approximately 25,000 die as a consequence each year, where the incidence in recent years involving an increasingly younger population is worrying, with 25% occurring between the ages of 20 and 64, and becoming more frequent from the age of 65 [4].

Globally, the prevalence of stroke is also rising. According to the Global Burden of Disease Study, in 2021, there were more than 101 million people living with the consequences of stroke worldwide, with a global prevalence rate of approximately 1.26% [5]. However, some regions of Europe report significantly higher prevalence rates, reaching up to 9.2% in older populations [6].

In high-income countries, cerebrovascular diseases have a significant slowdown in mortality [7], and there is a correlation between educational level and stroke risk [8]. However, it does not improve adequate knowledge about stroke prevention and management [9]. This gap in awareness highlights the need to emphasize effective preventive strategies in public health campaigns. Among these strategies, physical activity plays a key role as a modifiable lifestyle factor that reduces the risk of stroke.

Women who engage in regular physical activity have a protective factor against stroke of up to 70% [10]. A significant correlation was found between functional independence and quality of life in stroke survivors [11]. On the other hand, depending on gender approaches to treatment and rehabilitation may be different [12]. At the hormonal level, natural estrogens specific to women have anti-atherogenic and neuroprotective effects during the female reproductive years, generating a protective effect against atherosclerosis before menopause and a higher incidence of these diseases after menopause [13]. These hormonal differences may contribute to the observed disparities in stroke incidence, age of onset, and recovery between men and women, which underscores the importance of analyzing sex-specific patterns in prevalence, limitations, and access to rehabilitation.

After a stroke, motor recovery of the lower limbs has a greater impact on quality of life than motor recovery of the upper limbs after 60 days of neurorehabilitation [14]. In addition, alterations in the perception of stimuli affect 70% of survivors, causing distress in the interpretation of emotions and tasks and a greater dependence on others to understand proposals [6]. Stroke is mainly caused by modifiable risk factors such as hypertension, diabetes, dyslipidemia, smoking, sedentary lifestyle, and cardiovascular conditions, which makes prevention strategies especially relevant [15]. Stroke leads to long-term disability in many survivors, causing a significant deterioration in physical, cognitive, and emotional functioning and increasing dependency. It also represents a considerable economic burden on healthcare systems due to the high costs of acute care, rehabilitation, and long-term support services [16,17].

Taking the above into consideration, it is essential to consider that similar rehabilitation challenges and therapies are shared with other neurological disorders such as multiple sclerosis (MS). In people suffering from MS, cognitive and motor rehabilitation has shown promising effects on autonomy, emotional well-being, and quality of life. These parallels suggest that comprehensive, interdisciplinary rehabilitation approaches may have had positive effects across neurological conditions, including stroke [18,19].

For all these reasons, knowing the state of health, limitations, professional follow-up, and possible differences by sex in patients who have suffered a stroke is essential to establish measures, not only for prevention but also for long-term action and follow-up treatment. Despite advances in acute stroke care, current rehabilitation programs in Spain present important gaps, especially in long-term follow-up, continuity of physiotherapy, and access to multidisciplinary services—particularly in rural or underserved areas. We hypothesized that sex and sociodemographic characteristics significantly influence access to rehabilitation and the presence of functional limitations among stroke survivors in Spain. Therefore, the aim of this study was to analyze the prevalence of stroke and explore disparities in rehabilitation and quality of life related to these factors.

## 2. Materials and Methods

A cross-sectional study of the data collected in Spain by the latest European Health Interview Survey (EHIS) was conducted between 2019 and 2020 and published by the National Institute of Statistics (INE), whose main objective is to obtain information on different aspects related to the health of the Spanish population. This survey is coordinated by Eurostat and regulated by Regulation (EC) 1338/2008 and Commission Regulation 141/2013 [20].

The methodological procedure carried out to select the sample and determine its size, as well as the inclusion and exclusion criteria in the European Health Interview Survey, are detailed in the survey protocol [21]. For this study, cases with a medical diagnosis of stroke (including cases of embolism, cerebral infarction, and cerebral hemorrhage) in patients over 16 years of age were selected, regardless of whether the stroke occurred within the last year or prior. For these cases, sociodemographic variables (age, sex, level of education, cohabitation, and occupation), self-perception of health, limitations in carrying out activities of daily life, level of physical activity and sedentary lifestyle, and attendance were studied during the last year to a specialist physiotherapist.

In order to meet the objectives of this study, qualitative variables were analyzed using absolute frequencies and percentages. Prevalence was calculated as the quotient between the number of stroke cases found and the number of individuals in the sample. Quantitative variables were assessed using the mean and standard deviation. Hypothesis tests based on the chi-square distribution were performed to compare qualitative variables. In the case of quantitative variables, the Student’s test was used. Confidence intervals were constructed assuming normality in the data given the sample size used. In all cases, a significance level of 5% was assumed. IBM SPSS v.26 was used. In addition, the Epidat 4.2 program was used to construct the population pyramid of stroke cases.

## 3. Results

**Figure 1:**
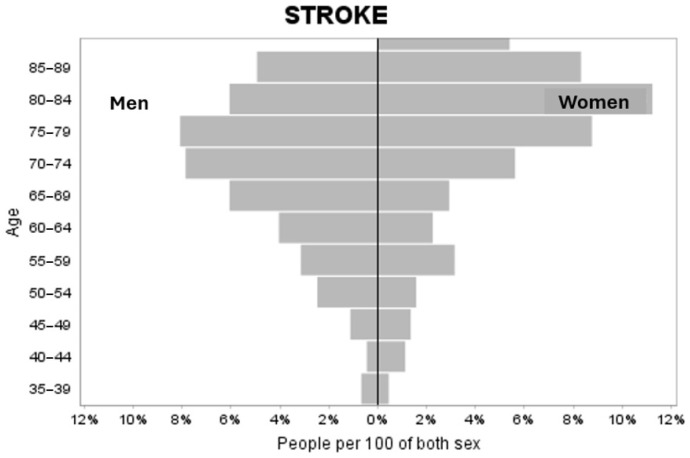
Distribution of the percentage of stroke by sex and five-year age groups.

The EES was performed in Spain on a total of 22,072 people aged between 15 and 104 years, with a mean age of 54.57 years (SD 19.01). Of the participants, 47.1% were men, indicating a lower proportion of males compared to females. Of this, 445 people had suffered a stroke, which represents a prevalence of 2.02% (CI(95%) = (1.83%; 2.2%)). Of those who had suffered a stroke, 46.3% were men. No statistically significant differences exist in the gender representation of the reference population. The ages ranged from 35 to 98 years, with a mean age equal to 73.95 (S.D. 12.82 years). A total of 25% of the sample was under 66 years of age, and 50% was under 73 years of age. Differentiating by sex, men had a mean age of 71.28 years (S.D. 12.0), with the minimum age being 36 years and the maximum 97. In women, the mean age was 76.26 years (S.D. 13.1), with 35 years as the minimum age and 98 as the maximum age, with these age differences being significant with *p* < 0.001. Fifty percent of the men were under 73 years of age and, in the case of women, under 79 years of age. Figure 1 shows the distribution of stroke cases by sex and five-year age groups.

With regards to the perception of one’s state of health in the last 12 months, it is women who report worse health status and more limitations and difficulties in carrying out daily activities, showing significant differences by sex. There are significant differences in educational attainment between men and women; however, no such differences were found when analyzing occupational status. Although some respondents report limitations due to mental health problems, physical limitations are the most prevalent among both men and women. Most of those affected by stroke suffer from chronic or long-term diseases. Detailed information on physical limitations and their differences between men and women is given in Table 1.

### 3.1. Physical Activity

Concerning the level of physical activity of patients who have suffered a stroke, 93.3% remained seated, performing their main activity or standing without making great movements or efforts. In addition, in their free time, most of the sample does not perform any type of physical activity. On average, men walk at least 10 min 4.21 days a week, while women walk at least 10 min 3.05 days a week. In addition, most of the respondents do not walk for more than 30 min, with only 14.1% of men walking for an hour or more and 12.1% of women walking for an hour or more. The average time a stroke victim spends sitting on any given day is between 9.09 and 10.98 h for men and women, respectively. In general, in terms of physical activity, no significant differences were found by sex. Detailed information on physical activity levels and their differences between men and women is provided in Table 2.

#### Specialist Contacts and Limitations

During the 12 months prior to the survey, 83.6% of the people who had suffered a stroke did not have an appointment with a rehabilitation specialist (physiotherapist). Despite the fact that, as shown in the previous table, sedentary behavior is similar in men and women, Table 3 shows that when it comes to carrying out everyday activities (such as getting dressed, going to bed, going to the toilet, preparing food, using the telephone, shopping, or taking a shower), men and women who have suffered a stroke show great differences.

## 4. Discussion

This study reveals the prevalence of stroke in Spain, estimated at 2.02% of the general population, depicts a figure much lower than the European prevalence of 9.2% IC(95%) [6].

Although the prevalence of stroke was similar between men and women, relevant differences were identified according to sex in aspects such as level of education, type of cohabitation and quality or style of life. The level of education and income, being employed and being healthy are independent factors of an “adequate knowledge” of stroke, so it is recommended that educational campaigns should be carried out in simple language, with special interest at the most vulnerable societal target group [9,22]. If the symptoms of stroke are not recognized, and citizens do not know how to realize the urgency, care will be delayed in the pre-hospital phase [23].

A less healthy lifestyle prior to a stroke is associated with a poorer post-stroke quality of life [24]. This study reveals that a significant percentage of the sample has limitations in activities of daily living despite the fact that more than 50% are under 76 years of age. This finding shows the early onset of functional difficulties in a relatively young population within the group of older people, which points to the importance of implementing programs that promote healthy aging. Various studies have shown that regular physical activity plays a key role in maintaining cognitive function and quality of life, helping to prevent the risk of needing care and deterioration associated with aging [25,26]. Among the most recommended exercises after stroke, we find the treadmill (to work different speeds and stride widths) and walking outdoors at low intensity [27]. Treadmill exercise has therefore been found to affect pathways associated with blood clotting, cartilage formation, and fat metabolism after a stroke [28]. In any case, physical activity remains a modifiable lifestyle factor that should be included in stroke prevention strategies in both women and men [10]. Physical activity will reduce the patient’s chances of experiencing fatigue, improving their quality of life [11].

Regarding the age of stroke presentation, men tend to suffer strokes at younger ages, while women at older ages, as observed in this study. This could explain why women report a poorer quality of life after the event, possibly due to increased dependence and poorer baseline fitness. Comparing these findings with previous studies, we found that the overall risk of stroke in women compared to men was higher in women than in men younger than 30 years, lower in women compared to men in middle age, and similar in women and men ≥ 80 years old [29]. These differences may be related to specific physiological factors in male and female patients. In the case of women, specific natural estrogens have anti-atherogenic and neuroprotective effects [13]. However, cellular responses to cerebral ischemia also respond mechanically differently, which also suggests that the molecular signaling pathways activated by ischemia may differ in the brains of both sexes [30].

Another concerning outcome and finding in the results of this study is that most patients have not received care from a rehabilitation specialist in the last year despite the high prevalence of functional limitations. It is crucial to know the most critical reasons for the delay in pre-hospital phase care. As other authors argued, the most common cause for the delay in care in the pre-hospital phase is that the symptoms are not recognized as typical signs of stroke, and the affected person is not aware of how to assess the urgency; in many cases, waits for symptoms to disappear; does not request help; or if they do, it is too late. This may be more serious among the most vulnerable social classes in rural areas and older subjects, given the low degree of knowledge acquired about strokes [24]. It has been shown that people with more education and a higher income level tend to seek medical attention faster when faced with the symptoms of a stroke [31].

Although a considerable percentage of people do not report severe limitations in walking or performing basic activities, a very high percentage lead a sedentary life. This sedentary behavior may originate both before and after stroke, representing either a contributing risk factor or a post-stroke consequence linked to limited mobility, fatigue, or reduced access to rehabilitation. This underscores the need to promote physical exercise as a preventive measure in the general population and post-stroke patients. We found one study where the benefit of regular physical activity in women and in some populations was associated with a reduction in stroke risk of up to 70% [10]. One study shows lower tendencies for depression, greater resilience, and mobility when performing aerobic physical activity such as running or walking outdoors [27].

These conclusions support previous evidence indicating the persistence of functional limitations after stroke, particularly in younger segments of the older population. While innovative rehabilitation strategies are being explored in clinical settings, such as virtual reality and telerehabilitation, our data highlight a more urgent need: improving access to basic post-stroke care and long-term physiotherapy. The limited use of rehabilitation services reported by participants reinforces the importance of strengthening conventional support systems, especially in vulnerable populations. It is also paramount to note that socioeconomic status may act as a confounding factor in access to rehabilitation services. Individuals with lower income or educational levels may face greater barriers to receiving continuous care, which could partially explain the disparities observed. Future studies should consider adjusting for these factors to better isolate the impact of sex, age, or geographic location.

Given that life expectancy in Spain exceeds 84 years, the health system must prioritize strategies that promote healthy aging, with special attention to patients who have suffered a stroke. It is essential to increase access to physiotherapy and other long-term rehabilitation services, as well as to raise awareness among professionals and patients about the benefits of maintaining active follow-up after a stroke.

### Limitations of the Study

The main limitation of this study is that it is a cross-sectional study that does not allow causal relationships to be established, although it does guide the search for these relationships in future research. On the other hand, we could assume a perception bias as some data are self-reported; however, we present a sufficiently large and representative sample of the general population that minimizes this bias subsequently. The questions related to limitations or health status in the last 12 months shows that despite the fact that most strokes occurred before the last year, the population has sequelae that must be taken into account to improve the continuous care of these people.

## 5. Conclusions

Stroke continues to be a relevant public health problem in Spain, with a marked incidence and impact that varies according to gender, which underlines the need for differential approaches in post-stroke care. At the same time, significant inequalities persist in access to information about stroke and specialized services, which influences the quality of the recovery process and equity in care. These challenges highlight the evident need for a comprehensive approach that combines prevention strategies, health education and equitable access to rehabilitation processes adapted to the needs of the entire population. In this context, strengthening health literacy and reducing socioeconomic barriers can significantly improve both stroke reaction time and long-term functional outcomes. Given the progressive aging of the population, it is a priority to guide future research towards the design of sustainable primary prevention strategies based on the community and adapted to the functional and social profile of each patient.

## Figures and Tables

**Table 1:** Sociodemographic information, health perception, and limitations.

		Men *n* (%)	Women *n* (%)	*p*-Value
Level of education	No studies or Incomplete Primary Education	34 (30.4)	78 (59.6)	<0.001
	Primary education	68 (47.9)	74 (52.1)	
	Secondary education	70 (49.7)	71 (51.3)	
	University studies	34 (68.0)	16 (32.0)	
Cohabitation	With spouse/domestic partner	136 (61.6)	84 (38.4)	<0.001
	Not living together as a couple	70 (31.1)	155 (68.9)	
Current or Past Occupation	Direction/management > 10 employees	17 (58.6)	12 (41.4)	0.356
	Direction/management < 10 employees	14 (50.0)	14 (50.0)	
	Intermediate occupations and self-employed	31 (43.7)	40 (56.3)	
	Supervisors and workers in technical occupations	40 (51.3)	38 (48.7)	
	Skilled workers in the primary sector and others	81 (50.6)	79 (49.4)	
	Unskilled workers	21 (36.8)	36 (63.2)	
Health status in the last 12 months	Very good	4 (36.4)	7 (63.6)	0.025
	Good	57 (60.6)	37 (39.4)	
	Fair	77 (43.8)	99 (56.3)	
	Bad	54 (43.5)	70 (56.5)	
	Very bad	14 (35.0)	26 (65.0)	
Have a chronic disease or health problem	Yes	198 (45.8)	234 (54.2)	0.263
	No	8 (61.5)	5 (38.5)	
Limitations on carrying out activities	Severely limited	56 (42.1)	77 (57.9)	0.021
	Limited, but not severely	86 (42.8)	115 (57.2)	
	Nothing limited	64 (57.7)	47 (42.3)	
Type of problem causing the limitations in performing activities	Physics	109 (43.8)	140 (56.2)	0.716
	Mental	4 (36.4)	7 (63.6)	
	Both	29 (39.2)	45 (60.8)	
Difficulty walking 500 m on flat ground without any help	No difficulty	119 (58.6)	84 (41.4)	<0.001
	Some difficulty	37 (35.9)	66 (64.1)	
	A lot of difficulty	26 (32.1)	55 (67.9)	
	I cannot do it at all	24 (41.4)	34 (58.6)	
Difficulty going up or down 12 steps	No difficulty	106 (63.1)	62 (38.9)	<0.001
	Some difficulty	43 (41.3)	61 (58.7)	
	A lot of difficulty	31 (30.7)	70 (69.3)	
	I cannot do it at all	26 (36.1)	46 (63.9)	
Difficulty remembering or concentrating	No difficulty	118 (52.9)	105 (47.1)	0.008
	Some difficulty	54 (40.9)	78 (59.1)	
	A lot of difficulty	30 (43.5)	39 (56.5)	
	I cannot do it at all	4 (19.0)	17 (81.0)	

**Table 2:** Level and frequency of physical activity.

		Men	Women	*p*-Value
Main activity in the workplace, school, home (housework)…	Sitting most of the day	118 (44.7)	146 (55.3)	0.956
	Standing most of the day without making large movements or efforts	46 (46.5)	53 (53.5)	
	Walking, carrying some weight, making frequent movements	11 (50.0)	11 (50.0)	
	Performing tasks that require great physical effort	2 (50.0)	2 (50.0)	
How often do you do any physical activity in your free time?	I do not exercise. I occupy my free time in an almost completely sedentary way (reading, watching television, going to the movies, etc.).	107 (42.0)	148 (58.0)	0.189
	I do some occasional physical or sports activity (walking or cycling, gardening, gentle gymnastics, recreational activities that require a light effort, etc.)	74 (51.7)	69 (48.3)	
	I do physical activity several times a month (sports, gymnastics, running, swimming, cycling, team games, etc.)	13 (50.0)	13 (50.0)	
	I do sports or physical training several times a week	12 (57.1)	9 (42.9)	
In a week of normal activity, how many days do you walk at least 10 min at a time to get around? *		4.21 (3.0)	3.05 (3.01)	<0.001
Usually, on one of those days, how long do you walk to get around?	10 to 29 min	81 (48.5)	86 (51.5)	0.095
	30 to 59 min	47 (61.0)	30 (39.0)	
	One hour or more but less than two hours	16 (50.0)	16 (50.0)	
	Two hours or more but less than three hours	4 (100)	0 (0)	
	Three hours or more	1 (100)	0 (0)	
In your leisure time during a normal activity week, how many days do you play sports, gymnastics, cycling, brisk walking, etc., at least 10 min at a time? *		1.31 (2.37)	1.19 (2.27)	0.303
In your leisure time during a normal activity week, how many days do you do activities specifically aimed at strengthening your muscles? *		0.39 (1.37)	0.3 (1.28)	0.233
How long (hours) do you sit on a typical day? *		9.09 (14.68)	10.98 (18.69)	0.121

* Mean values and standard deviation are expressed. The number of men is 206, and the number of women is 239.

**Table 3:** Main limitations in stroke survivors.

			Men	Women	*p*-Value
Difficulty doing activities without help	Feed	No difficulty	162 (47.4)	180 (52.6)	0.351
		Some difficulty	10 (31.3)	22 (68.8)	
		A lot of difficulty	7 (46.7)	8 (53.3)	
		I cannot do it by myself	6 (40.0)	9 (60.0)	
	Sitting, getting up from a chair or bed, lying down	No difficulty	145 (53.9)	124 (46.1)	<0.001
		Some difficulty	15 (24.2)	47 (75.8)	
		A lot of difficulty	12 (30.0)	28 (70.0)	
		I cannot do it by myself	13 (39.4)	20 (60.6)	
	Dressing and undressing	No difficulty	144 (53.7)	124 (46.3)	<0.001
		Some difficulty	14 (25.5)	41 (74.5)	
		A lot of difficulty	11 (30.6)	25 (69.4)	
		I cannot do it by myself	16 (35.6)	29 (64.4)	
	Go to service	No difficulty	149 (50.5)	146 (49.5)	0.009
		Some difficulty	12 (28.6)	30 (71.4)	
		A lot of difficulty	8 (28.6)	20 (71.4)	
		I cannot do it by myself	16 (41.0)	23 (59.0)	
	Showering or bathing	No difficulty	133 (56.6)	102 (43.4)	<0.001
		Some difficulty	22 (32.4)	46 (67.6)	
		A lot of difficulty	8 (21.1)	30 (78.9)	
		I cannot do it by myself	22 (34.9)	41 (65.1)	
Difficulty doing on their own and without help	Preparing meals	No difficulty	116 (49.8)	117 (50.2)	<0.001
		Some difficulty	8 (21.6)	29 (78.4)	
		A lot of difficulty	3 (16.7)	15 (83.3)	
		I cannot do it by myself	26 (33.3)	52 (66.7)	
	Use the phone (look up numbers, dial, …)	No difficulty	147 (51.2)	140 (4.8)	0.005
		Some difficulty	8 (29.6)	19 (70.4)	
		A lot of difficulty	7 (30.4)	16 (69.6)	
		I cannot do it by myself	18 (31.6)	39 (68.4)	
	Make purchases (food, clothes, …)	No difficulty	119 (56.7)	91 (43.3)	<0.001
		Some difficulty	15 (39.5)	23 (60.5)	
		A lot of difficulty	7 (18.4)	31(81.6)	
		I cannot do it by myself	31 (31.6)	67 (68.4)	
	Taking your medicines, including remembering how much and when to take them	No difficulty	148 (53.8)	127 (46.2)	<0.001
		Some difficulty	14 (35.0)	26 (65.0)	
		A lot of difficulty	1 (4.5)	21 (95.5)	
		I cannot do it by myself	21 (34.4)	40 (65.6)	
	Perform light household chores (doing laundry, making the bed, cleaning the house, …)	No difficulty	101 (55.5)	81 (44.5)	<0.001
		Some difficulty	15 (32.6)	31 (67.4)	
		A lot of difficulty	10 (20.4)	39 (79.6)	
		I cannot do it by myself	31 (33.0)	63 (67.0)	
	Occasionally performing household chores that require a great deal of effort (moving furniture, cleaning windows, …)	No difficulty	78 (66.7)	39 (33.3)	<0.001
		Some difficulty	18 (39.1)	28 (60.9)	
		A lot of difficulty	20 (40.0)	30 (60.0)	
		I cannot do it by myself	40 (26.3)	112 (73.7)	
	Manage your own money (pay bills, deal with the bank, …)	No difficulty	141 (53.2)	124 (46.8)	<0.001
		Some difficulty	11 (33.3)	22 (66.7)	
		A lot of difficulty	6 (26.1)	17 (73.9)	
		I cannot do it by myself	21 (29.2)	51 (70.8)	

## Data Availability

The data used in this study were obtained from the European Health Interview Survey (EHIS) conducted in Spain between 2019 and 2020 and published by the National Institute of Statistics (INE). The data are publicly available through the INE website at the following link: https://ine.es/dyngs/INEbase/es/operacion.htm?c=Estadistica_C&cid=1254736176784&idp=1254735573175 (accessed on 2 November 2024). The survey is coordinated by Eurostat and regulated by Regulation (EC) 1338/2008 and Commission Regulation 141/2013.
